# Disagreement between two common biomarkers of global DNA methylation

**DOI:** 10.1186/s13148-016-0227-0

**Published:** 2016-05-23

**Authors:** Claudia Knothe, Hiromi Shiratori, Eduard Resch, Alfred Ultsch, Gerd Geisslinger, Alexandra Doehring, Jörn Lötsch

**Affiliations:** Institute of Clinical Pharmacology, Goethe University, Theodor-Stern-Kai 7, 60590 Frankfurt am Main, Germany; Project Group Translational Medicine and Pharmacology TMP, Fraunhofer Institute for Molecular Biology and Applied Ecology IME, Theodor-Stern-Kai 7, 60590 Frankfurt am Main, Germany; DataBionics Research Group, University of Marburg, Hans-Meerwein-Straße, 35032 Marburg, Germany

## Abstract

**Background:**

The quantification of global DNA methylation has been established in epigenetic screening. As more practicable alternatives to the HPLC-based gold standard, the methylation analysis of CpG islands in repeatable elements (LINE-1) and the luminometric methylation assay (LUMA) of overall 5-methylcytosine content in “CCGG” recognition sites are most widely used. Both methods are applied as virtually equivalent, despite the hints that their results only partly agree. This triggered the present agreement assessments.

**Results:**

Three different human cell types (cultured MCF7 and SHSY5Y cell lines treated with different chemical modulators of DNA methylation and whole blood drawn from pain patients and healthy volunteers) were submitted to the global DNA methylation assays employing LINE-1 or LUMA-based pyrosequencing measurements. The agreement between the two bioassays was assessed using generally accepted approaches to the statistics for laboratory method comparison studies. Although global DNA methylation levels measured by the two methods correlated, five different lines of statistical evidence consistently rejected the assumption of complete agreement. Specifically, a bias was observed between the two methods. In addition, both the magnitude and direction of bias were tissue-dependent. Interassay differences could be grouped based on Bayesian statistics, and these groups allowed in turn to re-identify the originating tissue.

**Conclusions:**

Although providing partly correlated measurements of DNA methylation, interchangeability of the quantitative results obtained with LINE-1 and LUMA was jeopardized by a consistent bias between the results. Moreover, the present analyses strongly indicate a tissue specificity of the differences between the two methods.

## Background

Epigenetic effects are exerted by various factors such as early social experiences [1–3], physical training [4], age [5], nutritional or chemical factors such as royal jelly [6], benzene [7], asbestos, smoking [5], and drugs [8]. For example, drugs may interfere with epigenetics [8] including all classical mechanisms such as histone modifications, DNA methylation [9, 10], and further regulatory processes of gene expression [11, 12]. This interference can be intended as with epigenetic therapeutics or unintended as common drugs may also exert epigenetic (side) effects [8]. The consequences reach from none, via modulating the disease or introducing disease independent symptoms, up to a possible hereditability of epigenetic fingerprints provided that epigenetic transmission, so far only shown in plants [13], extends to animals. Moreover, the influences between drugs and epigenetics are mutual. Not only can drugs modulate epigenetics, but epigenetics is also increasingly recognized as a source of interindividual variability in drug effects [11]. Quantification of epigenetic modulations has therefore manifold applications [14].

Assessing global DNA methylation is a frequent used marker for epigenetic screening. It captures the DNA methylation also at unknown genetic locations while the results of average DNA methylation correlate with the methylation of some trait-relevant genes [15]. The gold standard in this field is HPLC quantification of the 5-methylcytosine content (methyl group added to the 5-carbon position of a cytosine) within the whole genome that occur mostly at CpG sites [16]. However, due to its high demand in DNA amount and the difficulty to apply the method in high throughput approaches [17], alternatives have been developed [18]. Widely accepted are (i) the cumulative analysis of methylation at CpG sites in repeatable elements such as long interspersed nuclear element 1 (LINE-1) [18] dispersed in more than 500,000 copies across the whole human genome [19–21] and (ii) the luminometric methylation assay (LUMA) of overall 5-^m^C content in “C^m^CGG” recognition sites across the whole genome [22].

The utility of both biomarkers is supported by the correlation of their results with the HPLC gold standard [23, 24]. However, both methods address different recognition sites within the genome. Specifically, while LINE-1 is overrepresented in A+T rich regions, LUMA recognition sites are more dense in C+G rich regions [25]. Nevertheless, both methods are widely used as markers of global methylation [26–36] as if they were equivalent. Consistent with their biological differences, however, doubts have been raised about such equivalence. Indeed, the two methods quantified the global DNA methylation in colon biopsies, cell lines [24], and human blood cells [37] differently. However, this was based on limited sample sizes [23, 24] and DNA methylation ranges [37]. Considering the increasing importance of the assessment of unknown epigenetic effects such as of drugs [8] or alimentary materials [14], where a definite set of target genes for such epigenetic effects can often not be predefined, the present study aimed at systematic assessment of the agreement between the two bioassays. With a clinical focus analogously to a previous investigation [38], the analyses were performed in human blood cells that are frequently the only easily available biological material in human studies [26–36]. However, to increase the range of global DNA methylation, human-derived cell populations (MCF7 and SHSY5Y cell lines) were added following treatment with methylation modifying substances. This provided a total of 238 samples. The underlying hypothesis of the present method comparison was the non-agreement between the two assays, as suggested by the biological differences of their recognition sites.

## Methods

### Study design and subjects

The two different global DNA methylation markers (LINE-1 pyrosequencing, LUMA) were assessed in three independent sample sets that were generated (i) in vitro from human cell lines (MCF7 and SHSY5Y) or (ii) whole blood samples acquired from healthy volunteers (one set) or (iii) pain patients (two sets). The in vitro approach served to induce a broad variation of DNA methylation as the required suitable basis for correlation analysis using known modulators of DNA methylation to the cell culture under highly controlled laboratory conditions. The LINE-1-based data had been analyzed previously in a non-redundant context addressing the effect of drug exposure on DNA methylation [39]. To picture the clinical setting, whole blood-derived DNA collected from either healthy subjects or chronic pain patients was analyzed. The in vivo assessments followed the Declaration of Helsinki and were approved by the Ethics Committee of the Goethe University, Frankfurt am Main, Germany. Informed written consent from each participating subject had been obtained. The actual health status of the healthy volunteers was ascertained by medical history and physical examination including vital signs. Exclusion criteria were a current clinical condition, any other actual diseases, and drug intake within a week except oral contraceptives. Patient’s samples were available from a previous assessment of chronic pain patients treated with either opioid or non-opioid analgesics [38].

### DNA sample acquisition

#### Human blood samples

DNA from whole blood samples and opioid-related phenotypes was available from previous studies [40–42]. Cohort 1 consisted of 83 (26 men, 57 women, aged 39.6 ± 7.02) healthy subjects that were drawn randomly from a control cohort. Cohorts 2 and 3 comprised pain patients in tertiary care. Cohort 2 contained 29 (14 men, 15 women, aged 47.8 ± 7.36) pain patients with an opioid treatment duration of ≥1 year and an average daily opioid dose of 52.69 ± 22.11 mg of oral morphine equivalents [43, 44]. Cohort 3 consisted of 19 (2 men, 17 women, aged 45.7 ± 11.63) pain patients who had received no opioids during their analgesic therapy (Tables 1 and 2). Sex and age matching was not possible due to insufficient human material for LINE-1 and LUMA assessments each in two independent measurements.

**Table 1:** Samples, conditions, and concentrations used for the assessment of methylation levels by means of LINE-1 and LUMA, of which the cell lines had been obtained previously [39]

Data subset	Tissue (cell type)	Age (mean ± SD) (years)	*n* (total number of replicates)	Condition	Treatment duration	Concentrations
MCF7	Human breast cancer cell line	–	6	Untreated	3 days	–
			7	DMSO	3 days	0.1 (%)
			9	5-Aza-CdR	3 days	0.1/0.3/1 (μM)
			7	SAM	3 days	10/50/100 (μM)
			5	DCP	3 days	0.1/1/10 (mg/l)
			11	Methadone	3 days	1/10/25/50/75/100 (μM)
			6	RG108	3 days	10/50/70/100 (μM)
			4	5-Aza-CdR + SAM	3 days	0.3 + 50/100 (μM)
			2	5-Aza-CdR + DCP	3 days	0.3 (μM) + 10 (mg/l)
			9	5-Aza-CdR + Methadone	3 days	0.3 + 10/25/50/75/100 (μM)
			2	5-Aza-CdR + SAM + DCP	3 days	0.3 (μM) + 100 (μM) + 10 (mg/l)
			2	RG108 + DCP	3 days	100 (μM) + 10 (mg/l)
			3	SAM + DCP	3 days	100 (μM) + 10 (mg/l)
SHSY5Y	Human neuronal cell line	–	7	Untreated	3 days/7 days	
			7	DMSO	3 days/7 days	0.1 (%)
			12	5-Aza-CdR	3 days/7 days	0.1/0.3/0.5/1 (μM)
			1	SAM	3 days	30 (μM)
			3	Methadone	3 days	10/100 (μM)
			1	5-Aza-CdR + SAM	3 days	0.3 + 30 (μM)
			3	5-Aza-CdR + Methadone	3 days	0.3 + 10/100 (μM)
Human blood	Healthy subjects	39.57 ± 7.02	83	Untreated	–	
	Pain patients, opioid treated	47.83 ± 7.36	29	Opioid analgesics	>1 year	52.69 mg ± 22.11 OME^a^
	Pain patients non-opioid treated	45.68 ± 11.63	19	Non-opioid analgesics		

^a^Oral morphine equivalents; opioid doses were converted to daily oral morphine equivalents (OME) using previously published conversion factors [43, 44]

**Table 2:** Descriptive and inference statistics of the two assays

Descriptives			Paired tests		Method of 95 % limits of agreement between measurements (“Bland-Altman”)					
Data subset (cell type)	LINE-1 (mean ± SD and (range)) (%)	LUMA (mean ± SD and (range)) (%)	Wilcoxon signed rank test	Spearman Correlation (95 % CI)	Mean difference (fixed bias) (95 % CI)	Significance of mean deviation from 0	KS-test of normal distribution of differences	95 % confidence limits of agreement	Slope (proportional bias) (CI of estimate)	Significance of deviation of slope from 0
MCF7	60.96 ± 11.99 (34, 76.18)	67.8 ± 11.99 (34.36, 78.34)	*V* = 2476, *p* = 6.22 × 10^−10^	*ρ* = 0.58 (0.38, 0.74), *p* = 5.53 × 10^−8^	−6.8 (−8.3, −5.3)	1.93 × 10^−13^	*D* = 0.1183, *p* = 0.24	−19.5, 5.8	−0.00046 (−0.13, 0.13)	0.99
SHSY5Y	59.01 ± 11.54 (31.91, 71.65)	55.66 ± 11.99 (24.89, 67.65)	*V* = 74, *p* = 4.66 × 10^−5^	*ρ* = 0.8 (0.56, 0.92), *p* = 1.31 × 10^−8^	3.3 (1.9, 4.8)	3.16 × 10^−5^	*D* = 0.068241, *p* = 0.99	−4.6, 11.3	−0.04 (−0.16, 0.08)	0.53
Human blood	92.4 ± 2.75 (77.9, 98.8)	72.3 ± 3.1 (61.9, 78.5)	*V* = 0, *p* <2.2 × 10^−16^	*ρ* = 0.35 (0.18, 0.51), *p* = 3.25 × 10^−5^	20.1 (19.5, 20.7)	3.41 × 10^−103^	*D* = 0.086144, *p* = 0.29	13.4, 26.8	−0.2 (−0.44, 0.045)	0.11

*KS-test* Kolmogorov-Smirnov test

#### Cell culture and exposure to known modulators of DNA methylation

Since the human blood samples did not provide sufficient variability in DNA methylation for the present analyses, human cell lines were added in which the desired broad range of methylation could be induced by treating the cells with known modulators of DNA methylation. Two human cell lines were chosen, i.e., MCF7 cells that is a breast cancer-derived cell line and SHSY5Y cells that possess a neuronal character. The choice was based on the criteria (i) human origin consistent with the present clinical focus, (ii) reported ability to respond with decreased DNA methylation to the stimulation with the demethylating agent 5-Aza-2′-deoxycytidine (5-Aza-CdR), which can be inhibited by simultaneous treatment with *S*-adenosyl methionine (SAM).

MCF7 cells were cultured in Dulbecco’s modified Eagle’s medium (DMEM) + GlutaMax™ (Gibco, Darmstadt, Germany) supplemented with 10 % (*v*/*v*) fetal calf serum (FCS) and 1 % penicillin/streptomycin (PAA, Cölbe, Germany). SHSY5Y cells were obtained from the DZMS Collection of Microorganisms and Cell Cultures (Braunschweig, Germany) and were grown in 1:1 mixed Ham F12 and Minimum Essential Medium (MEM) (Gibco, Darmstadt, Germany) supplemented with 15 % (*v*/*v*) FCS, 1 % Minimum Essential Medium Non-Essential Amino Acids (MEM NEAA, Gibco, Darmstadt, Germany), 2 mM L-glutamine (Gibco, Darmstadt, Germany), and 1 % penicillin/streptomycin (PAA, Cölbe, Germany) at 37 °C in humidified atmosphere containing 5 % CO_2_. In prior to drug treatment, cells were seeded at a density of 3 × 10^5^/10 cm^2^ and allowed to settle for 24 h in the complete media. Subsequently, cells were incubated for 72 h (MCF7, SHSY5Y) or 7 days (SHSY5Y) in the presence of the known or potential modulators of DNA methylation at various concentrations Table 1.

Drug treatment conditions have been reported elsewhere [39]. In brief, 5-Aza-2′-deoxycytidine (5-Aza-CdR), a cytidine analogue, covalently traps DNMTs, and RG108, a specific DNMT inhibitor, directly blocks the active site of the enzyme which are expected to lead to global DNA hypomethylation [45–48]. *S*-adenosyl methionine (SAM) is a methyl donor that is catalyzed by DNMTs to form 5-methyl cytosine at CpG sites [49], thereby, it is expected to increase DNA methylation or at least inhibit global hypomethylation induced by 5-Aza-CdR [50]. 2,4-Dichlorophenol (DCP) is an environmental pollutant reported to increase global methylation [51]. Methadone was chosen as opioid because it had been involved in the largest group of patients (heroin addicts) in whom the clinical association of opioid-induced hypermethylation had been observed [38]. On every day, media were replaced and compounds were added freshly. Methadone hydrochloride (Fagron, Barsbüttel, Germany) was dissolved in Dulbecco’s phosphate-buffered saline (DPBS) without CaCl_2_ and MgCl_2_ (Gibco, Germany, Darmstadt; 14190-094). 5-Aza-CdR, SAM, DCP (Sigma-Aldrich, Taufkirchen, Germany), and RG108 (Biomol, Hamburg, Germany) were dissolved in DMSO and mixed with solvent to obtain a final concentration of 0.1 % DMSO (0.25 % for RG108) to the cell media during incubation. Cells incubated with 0.1 % solvent alone or without any substance addition (i.e., the control condition) served as controls.

### Quantification of global DNA methylation

#### DNA isolation

Genomic DNA was extracted from cell line materials and whole blood samples with the DNeasy Blood and Tissue Kit (Qiagen, Hilden, Germany) according to the manufacturer’s protocol and eluted in water. Genomic DNA obtained from blood samples was concentrated using vacuum rotation (45 °C for 25 min) to reach at least a final concentration of 50 ng/μl.

#### Methylation analysis of retrotransposon LINE-1

The analysis of LINE-1 DNA methylation was performed identically as described previously in full detail [18]. Bisulfite treatment was performed using the EZ DNA Methylation-Gold Kit (Zymo Research, Freiburg, Germany) with 0.5–1 μg genomic DNA as instructed by the manufacturer.

The analyzed region of a CpG island located in the promoter region (L1Hs) DNA (PubMed GenBank X58075.1; lower strand) has the bisulfite-converted sequence 5′-TTTTG*AGTTAGGTGTGGGATATAGT*TT**YG**TGGTG**YG**T**YG**TTTTTTAAGT**YG**GTTTGAAAAGCTAATATTCGGGTGGGAGTGATTCGATTTTTTAGGTGCGTTCGTTATTTTTTTTTTTGATTCGGAAAGGGAATTTTTTGATTTT-3′ where the 146-bp PCR product contains four analyzed CpG methylation sites (bold) and annealing sites for the PCR primers (underlined) and the sequencing primer (italic), respectively [7, 52]. PCR reactions were run on a Mastercycler nexus gradient flexlid device (Eppendorf, Hamburg, Germany) in a 50-μl reaction volume including 5-μl bisulfite-treated DNA, mixed with 0.5 μl MyTaq™ HS DNA Polymerase (5 U/μl) (Bioline, Luckenwalde, Germany), 10 μl 5× MyTaq Reaction Buffer, 0.2 μl of each PCR primer (100 μM), and 34.1 μl HPLC-purified water. The following PCR program was used: 95 °C for 1 min, 40 amplification cycles at 95 °C for 15 s, 56 °C for 15 s, 72 °C for 15 s, and a final elongation step at 72 °C for 5 min.

The analysis of the global methylation marker LINE-1 was done by means of Pyrosquencing™ (Qiagen, Hilden, Germany) as described previously [7, 38, 52]. In brief, 50 μl of the PCR templates were processed and purified with the PyroMark Vacuum Prep Worktable (Biotage, Uppsala Sweden) and subsequently annealed to the sequencing primer (5′-AGTTAGGTGTGGGATATAGT-3′) at 80 °C for 2 min as instructed by the manufacturer.

Sequence analysis took place on a PSQ 96 MA System using the PyroMark Gold Q96 Reagents (Qiagen, Hilden, Germany) with the sequence to analyze TTYGTGGTGYGTYGTTTTTTAAGTYGGTTT. Pyro Q-CpG methylation software (version 1.0.9) had been used to determine the nucleotide dispensation order (ATCAGTGTGTCAGTCAGTCTAGTCTG). LINE-1 methylation values represent the mean percentage methylation across all four CpG sites, which were measured in duplicate samples within one run. In addition, each sample was measured in two independent runs, which were subsequently averaged.

The accuracy of the analyses was verified by adding positive and negative control samples. Specifically, each run included control DNA from the EpiTect PCR Control DNA Set (Qiagen, Hilden, Germany) that contained both bisulfite-converted 100 % methylated and completely unmethylated DNA as positive controls and unconverted unmethylated DNA as negative control. The bisulfite-converted methylated control DNA reached on average 75.08 ± 0.68 % methylation while the bisulfite-converted unmethylated control DNA reached only 3.37 ± 0.21 % methylation, which agrees with published values [18]. The negative PCR control did not show specific spikes for any injected nucleotide, which demonstrated assay specificity. All absolute methylation values were subsequently calibrated to the methylated and unmethylated control DNA to cover a range from 0 to 100 %. Non-CpG cytosine residues were used as built-in controls to verify the bisulfite conversion. The acceptable percentages for passed and checked quality were adjusted to the complete bisulfite-converted controls supplied by Qiagen. Samples not meeting the criteria for complete bisulfite conversion or pyrosequencing™ quality control checks were excluded. The interassay coefficients of variation for duplicates were 2.38 % for cell-line samples and 1.18 % for blood samples.

#### Luminometric methylation assay

The luminometric methylation assay (LUMA) was performed as described previously [22] with modifications previously proposed [24]. A common used isoschizomer pair to investigate global DNA methylation pattern is HpaII and MspI; HpaII digestion is inhibited if the internal cytosine is methylated (CmCGG) at recognition site whereas MspI is insensitive to CpG methylation within this sequence [37]. DNA methylation level is defined as the HpaII/MspI ratio that would be 1.0 if the DNA is completely unmethylated and would approach zero if the DNA is completely methylated [22, 53]. Because of reported star activity of EcoRI [24], we used MfeI, a methylation-insensitive restriction enzyme, as normalization reference. Four hundred to five hundred nanograms of genomic DNA was cleaved with either HapII + MfeI or MspI + MfeI in two separate 20 μl reactions containing 2 μl of 10× Tango Buffer (330 mM Tris-acetate, 100 mM Mg-acetate, 660 mM K-acetate, 0.1 mg/ml BSA, Thermo Scientific, Schwerte, Germany), 5 U of HpaII (10 U/μl; NEB, Frankfurt, Germany) or MspI (20 U/μl; NEB, Frankfurt, Germany), and 2.5 U of MfeI (10 U/μl; NEB, Frankfurt, Germany) at 37 °C for 16 h using a PSQ 96 Plate Low (Qiagen, Hilden, Germany). The incubation time could be reduced to 4 h without impacting the completion of the enzymatic reaction. Subsequent to digestion, 20 μl of annealing buffer (Qiagen, Hilden, Germany) was added to the cleavage reactions and samples were assayed in duplicate using the PSQ 96 MA System (Biotage AB, Uppsala, Sweden) and PyroMArk Gold Q96 reagents (Qiagen, Hilden, Germany). The sequence AC/TCGA was analyzed in SNP mode with ACTCGA nucleotide dispensation order. The dispensation order of dNTPs were dATPαS (step 1); mixture of dGTP + dCTP (step 2); dTTP (step 3); mixture of dGTP + dCTP (step 4); water (step 5); and dATPαS (step 6). Peak heights were calculated using the PyroMark™ ID software and HpaII/MfeI and MspI/MfeI ratios were determined as (dGTP + dCTP)/mean(dATP,dTTP) for each reaction. The HpaII/MspI ratio was then calculated as (HpaII/MfeI)/(MspI/MfeI), and methylation level was obtained as Methylation (%) = (1-HpaII/MspI) × 100. Samples with peak heights <2 (blood samples) or <1 (cell samples), MspI/MfeI ratio >4.2, and peak heights at dispensation peak 6 of more than 25 % relative to dispensation peak 1 were excluded from the analysis (modified [54]). The interassay coefficients of variation for duplicates were 3.28 % for cell samples and 2.17 % for blood samples.

The accuracy of the analysis was verified by including in each run an unmethylated probe of lambda phage DNA as 0 % control and a completely methylated probe of lambda phage DNA as 100 % control. All absolute methylation values measured in the three subsets of DNA samples, respectively, in human cell types were calibrated to the methylated and unmethylated lambda phage DNA control to cover the range of 0–100 %.

### Data analysis

The data analysis employed several bioinformatics methods to assess the agreement between the percentages of DNA methylation quantified by the LINE-1- or LUMA-based method. The analyses cover and extend previously proposed approaches to the statistics for laboratory method comparison studies [55]. It included (i) standard analysis of variance and correlation, (ii) visual inspection, (iii) the method of 95 % limits of agreement between measurements by two methods, (iv) Gauss mixture modeling, and (v) linear regression.

#### Analysis of variance and correlation, visual inspection

The first four analyses were performed using the R (version 3.2.1 for Linux; http://CRAN.R-project.org/) and SPSS (version 23 for Linux, IBM SPSS Statistics, Chicago, USA) software environments on an Intel Xeon® computer running on Ubuntu Linux 14.04 64-bit. In a first analytical approach, differences between DNA methylation assessed either by LINE-1 methylation or by LUMA were explored by submitting the data to analysis of variance for repeated measures (rm-ANOVA). “LINE-1/LUMA” was taken as within-subject factor and “data subset” (*n* = 3, Table 1) as between-subject factor, with post hoc Wilcoxon signed rank test-based [56] exploration of single differences. The *α* level was set at 0.05 and corrected for multiple testing according to Bonferroni [57]. Additional statistics included nonparametric correlation analyses calculating Spearman’s *ρ* [58], for which 95 % confidence intervals (CI) were obtained using 1000 bootstrap resamplings [59]. This was followed by the second approach, visual inspection of the scatter plot of the data and its placement relative to the line of equality.

#### Assessment of method agreement and bias

Absent correlation would discourage an agreement between the two assays. However, as pointed out previously, correlation analysis assesses the degree of association rather than the agreement between the methods and is insensitive to a possible bias [60]. Therefore, a third analytical approach employed the method of 95 % limits of agreement between measurements by two methods proposed by Bland and Altman [61]. For each data subset, differences in DNA methylation between the LINE-1- and LUMA-obtained magnitudes of DNA methylation were plotted against the mean of the two measurements (Fig. 3). The mean difference was an estimate of the fixed bias and tested for significant deviation from 0 on the basis of a one-sample *t* test. The 95 % confidence interval of the differences marked the limits of agreement for the two methods. A linear regression of the difference between the methods against their average indicated a relationship of the discrepancies between the measurements and the true value, which in the case of a slope significantly differing from 0 denoted the proportional bias. Calculations were performed using the R packages “BlandAltmanLeh” (B. Lehnert, https://cran.r-project.org/web/packages/BlandAltmanLeh/index.html) and “epade” (A. Schulz, https://cran.r-project.org/web/packages/epade/). Normality of the distribution of the differences between the two methods was assessed by means of Kolmogorov-Smirnov tests [62].

#### Pattern analysis of interassay differences

A data-subset specificity of the above differences was explored by fitting a mixture of Gaussian distributions (Gaussian mixture model (GMM) to their empirical distribution (Pareto density estimation (PDE)[63]) as given by the equation

1 $$ p(x) = {\displaystyle {\sum}_{i = 0}^M}{w}_iN\left(x\Big|{\mathrm{Mean}}_i,S{D}_i\right) = {\displaystyle {\sum}_{i = 1}^M}{w}_i\cdot \frac{1}{\sqrt{2\cdot \pi \cdot S{D}_i}}\cdot {e}^{-\frac{{\left(x-{\mathrm{Mean}}_i\right)}^2}{2\cdot S{D}_i^2}} $$

where *N(x|*Mean_*i *_*, SD*_*i*_*)* denotes Gaussian probability densities with means, Means_*i *_, and standard deviations, *SD*_*i *_, while the *w*_*i*_ is the mixture weights indicating the relative contribution of each component Gaussian to the overall distribution, and *M* denotes the number of components in the mixture. GMM fitting was performed with our R package “AdaptGauss” (M. Thrun, https://cran.r-project.org/web/packages/AdaptGauss/index.html; [64]), using the root mean square error between PDE and GMM as the fit criterion. The limits between the different Gaussians are defined by Bayes decision boundaries [65], i.e., the probability a data point being assigned to a specific Gaussian was calculated by an application of Bayes’ theorem [66], and the resulting grouping of the data was subsequently explored for association with data subsets, respectively, tissues, by applying a decision-tree algorithm [67] that used the information index, *f*(*p*) = − *p* ⋅ ln(*p*)_,_ to find optimal (local) dichotomic decisions. The method is invariant under transformations of the variables, robust with respect to outliers, and allows estimation of the misclassification rate [68]. The resulting tree model was cross-validated using a leave-*k*-out approach, where *k* was a randomly picked tenth of the total sample and the tree models were built 100 times with the respective remaining data. Calculations were done using the “rpart” function of the similarly named R package (B. Ripley; https://cran.r-project.org/web/packages/rpart/index.html).

#### Regression approach

The tissue-dependent relation between LINE-1 and LUMA measurements was further explored in a fifth analytical approach that employed linear modeling performed with the non-linear mixed effects modeling (NONMEM) software (version 7.3, Icon, Dublin, Ireland [69]). The analysis searched data-subset specific deviations from a y-intersection of zero and a slope of the value of one of the linear model expressed by extending its reduced form of

2 $$ {\mathrm{Methylation}}_{\mathrm{LINE}1} = \mathrm{Intersection} + \mathrm{Slope} \times {\mathrm{Methylation}}_{\mathrm{LUMA}} $$

to

3 $$ {\mathrm{Methylation}}_{\mathrm{LINE}1} = \left(\mathrm{Intersection} + {\theta}_{\mathrm{Int},\mathrm{Subset}1..\mathrm{Subset}3}\right)+\left(\mathrm{Slope} \times {\theta}_{\mathrm{Slope},\mathrm{Subset}1..\mathrm{Subset}3}\right) \times {\mathrm{Methylation}}_{\mathrm{LUMA}} $$

where

*θ*_Int,Subset1.. Subset3_ and *θ*_Slope,Subset1.. Subset3_

The *θ*s were allowed to take values differing from 0 or 1, for intersections and slopes, respectively, for each specific data subset while the values of the *θ*s were fixed at values of 0 or 1, respectively, for all other subsets. For example, for the MCF7 cell line, *θ*_Int,Subset1_and *θ*_Slope,Subset1_ described the deviations of the linear relationship from the other samples. The parameters were fit only for the MCF7 data while they remained fixed at 0 or 1 when other data was analyzed. The full linear model thus consisted of eight structural parameters *θ*, of which *θ*_1_ and *θ*_2_ denoted the global intersection and slope of the linear relationship, and *θ*_3..8_ accounted for the set-specific deviations from this global relationship and an additive residual error modeled as Methylation_Observed_ = Methylation_Predicted_ + *ε*, in which *ε* is a parameter with a mean of zero and a variance of *σ*^2^.

During the fitting process, parameters *θ*_Int,Subset1.. Subset3_ and *θ*_Slope,Subset1.. Subset3_ were introduced into the model in a stepwise fashion. Whether or not a specific θ remained part of the final model was established based on goodness-of-fit assessments, i.e., Occam’s razor or the principle of parsimony was applied. The simpler model was preferred to the more complex model as long as an additional parameter did not provide a significantly better fit. The main test was a likelihood ratio test, and therefore, the indicator of improvement of the fit was a change in minus twofold the log likelihood (Δ_−2LL_), and the *χ*^2^ approximation with the number of degrees of freedom equal to the difference in the number of parameters between two models was applied to judge statistical significance. Thus, the full model included an additional term and the reduced model involved the fixing of the respective term to a neutral value, i.e., 1 for factors and exponents and zero for summands. The *α* level was set at 0.05, which implies a significance criterion of Δ_−2LL_ <-3.84; for further details of the fitting process refer to [69]. Calculations were performed using “first order conditional estimation” [69].

Confidence intervals (95 %) of parameter values were calculated from 1000 runs of the final model with data sets that were obtained by bootstrap resampling [59] from the original data set [70], using PDxPop (version 5.10, Icon, Dublin, Ireland) for NONMEM. The limits of the 95 % confidence intervals of the parameter estimates were obtained as the 2.5th and 97.5th percentiles of the results of the 1000 model runs.

## Results

**Fig. 1:**
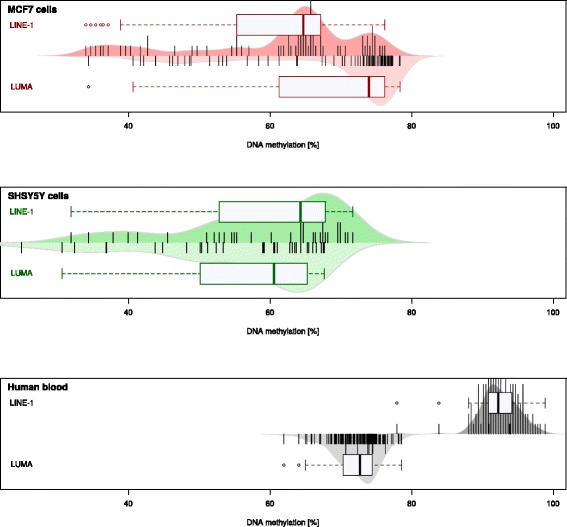
Raw observations and distribution of the global DNA methylation. The *beanplots* [91] show the single observations as stacked small lines in a one-dimensional scatter plot, surrounded by the probability density function (pdf) of the distributions. *Each panel* displays a single subset of the data (Table 1). It is composed of two beanplots of which the *upper* shows the raw methylation data based on the LINE-1 assay (*dark colored*, with different color of each data subset) and the *lower* shows the data based on the LUMA assay (*light colored*). *Box* and *whisker plots* of the identical data are overlaid on the beanplots. They have been constructed using the minimum, quartiles, median (*solid line within the box*), and maximum. Outliners are shown as points

**Fig. 2:**
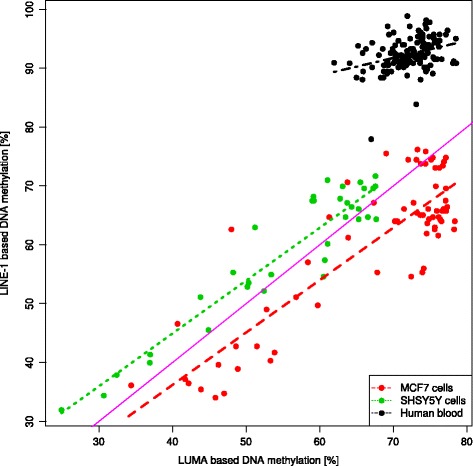
*Scatterplot* of the raw measurements (*n* = 238, Table 1) of global DNA methylation using the LINE-1 (ordinate) vs. the LUMA (abscissa) based bioassays, differently colored for single data subsets. The *solid magenta line* marks identity, and the *dashed* or *dotted lines* colored as the respective data show the results of the linear regression analysis for each data subset (Table 1)

**Fig. 3:**
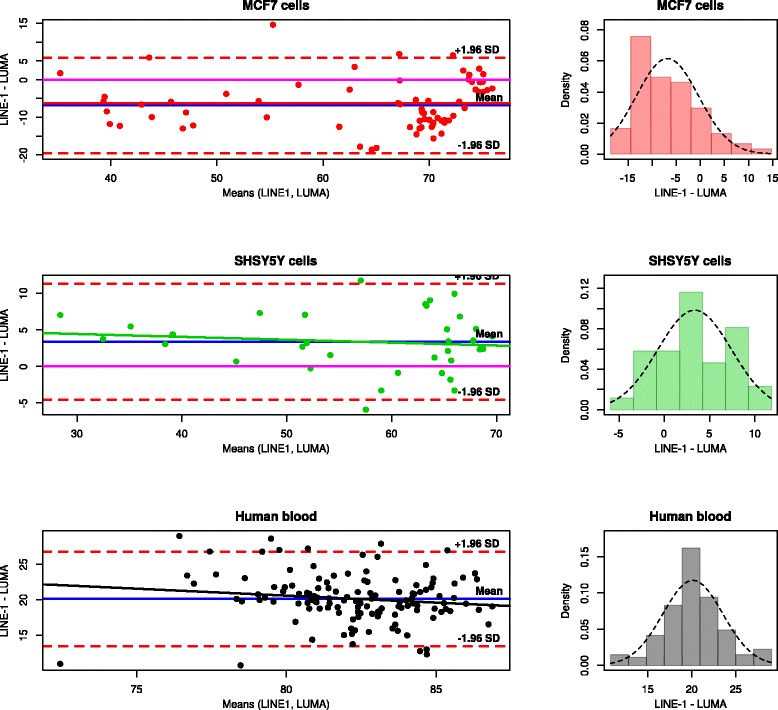
Plots of the differences between the measurements of DNA methylation using the LINE-1 and the LUMA based bioassays, for respective data subsets. *Left*: plots of the difference between the means of the two techniques (Bland and Altman plots [60]). *Each dot* illustrates a single difference. The fixed bias is represented by the gap between the *X* axis, corresponding to a zero difference (*magenta solid line*) and a *solid blue line* parallel to the *X* axis. The limits of agreement are indicated by the *red dashed lines* that limit the 95 % confidence interval (±1.96 standard deviations) of the measurement differences on either side of the mean difference. The proportional bias is indicated by a *solid trend line* in the same color as the data points. *Right*: distribution histogram of the differences between the measurements of the two assays. The *dashed line* represents normal distribution. Kolmogorov-Smirnov test for normal distribution accepted normality (*p* > 0.05). The plots were drawn using the “epade” (A. Schulz, https://cran.r-project.org/web/packages/epade/) package

**Fig. 4:**
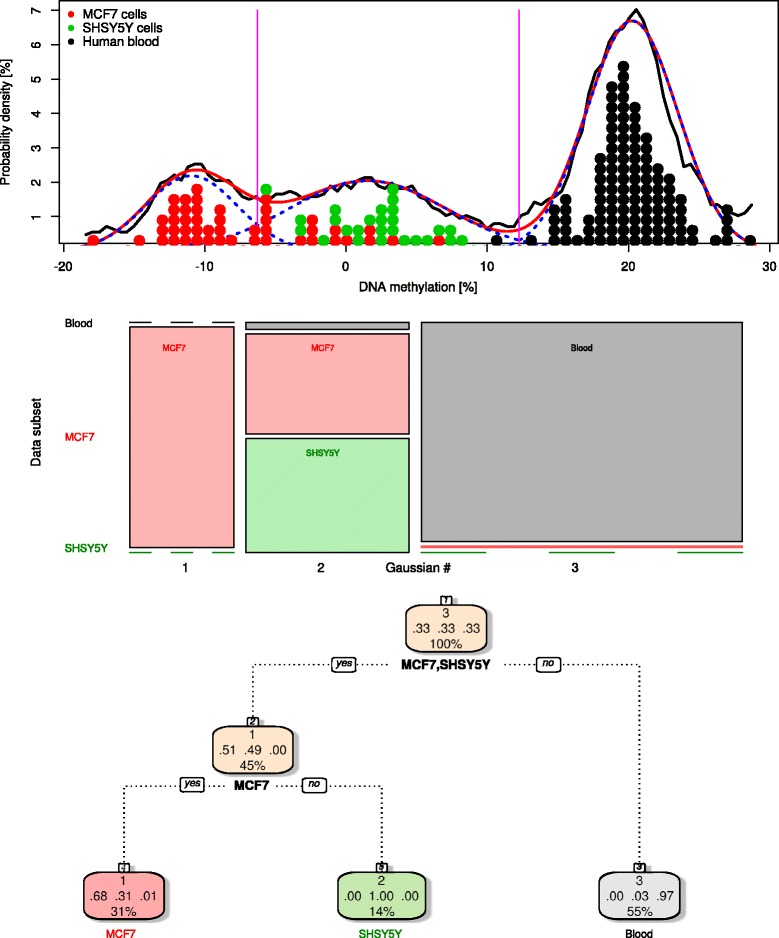
Pattern of differences between the measurements of DNA methylation using the LINE-1 and the LUMA based bioassays. *Top*: distribution of the differences observed in a total of *n* = 238 samples. Single differences are shown as *colored dots* matching the three data subsets (Table 1). The density distribution is presented as probability density function (PDF), estimated by means of the Pareto density estimation (PDE [63]; *black line*). A Gaussian mixture model (Eq. 1; GMM) was fit (*red line*) to the data, for which the number of mixes was *M* = 3 (*blue dotted lines*). The Bayesian boundaries between the three Gaussians are indicated as *magenta vertical lines. Middle*: mosaic plot showing the unequal distribution (*χ* ^2^ test: *p* < 2.2 × 10^−16^) of the data subset specific interassay differences (ordinate) among the three Gaussians (abscissa). The width of each cell is proportional to the number of measurements it comprises. *Bottom*: decision-tree showing the hierarchical criteria of assignment of an interassay difference to a Gaussian based group based on the originating tissue, i.e., data subset. The derived algorithm associated the majority of data from MCF7 cells, SHSY5Y cells, or blood cells to different Gaussians in the form of the following: “If the analyzed tissue consists not of cell lines (MCF7, SHSY5Y), then the LINE-1-LUMA differences belong to Gaussian 3 (counted from left to right refer to Fig. 4), and else, if the cell line is MFC7, then the differences belong to Gaussian 1, else they belong to Gaussian 2.” The model provided correct assignment at a cross-validated accuracy 83.6 %. *Three numbers in the middle of the nodes* display the proportion of single interassay differences in that node that really belonged to Gaussian #1, #2, or #3. At the *bottom of each node* is the percentage of data belonging to this node from all data (rounded to integer). The plot of the tree was obtained using the “fancyRpartPlot” function of the R package “rattle” (G. Williams; https://cran.r-project.org/web/packages/rattle/index.html [92])

Five applied methods were consistent with finding a disagreement between the magnitudes of DNA methylation measured with the LINE-1 or the LUMA method (Fig. 1). The first analytical approach, standard analysis of variance, identified differences between the assays that additionally differed among the three data subsets. A second approach was visual in nature. By looking at the scatterplot in Fig. 2, it is clear that the majority of DNA methylation values measured by the LINE-1 sequence lay above or below the line of equality. The third approach, the Bland and Altman method (Fig. 3), verified this observation by finding a fixed bias in all three data subsets. This in its extent additionally appeared to differ among data subsets. The fourth analytical approach (Fig. 4), Gauss mixture modeling (GMM), found groups of interassay differences that partially allowed identifying the underlying tissue. The fifth analytical approach, linear regression (Fig. 2), substantiated the observation of a tissue dependency of the relationship between the measurements taken by the two methods. The results of the different analyses will be reported in detail in the following.

### Visual inspection and analyses of variance and correlation

The distribution of the global DNA methylation of human blood, MCF7 cells, and SHSY5Y cells indicated differences between the two methylation markers LINE-1 and LUMA (Fig. 1). In MCF7 cells, the global DNA methylation appeared to be slightly smaller when assessed using LINE-1 than when assessed using LUMA. In the other data subsets, the opposite was observed, i.e., DNA methylation appeared to be slightly larger when assessed using LINE-1 than when assessed using LUMA. Data-subset specific interassay differences were substantiated by significant main effects of the rm-ANOVA factors “data subset” (*df* = 2,235, *F* = 200.11, *p* = 1.81 × 10^−51^) and “LINE1/LUMA” (*df* = 1,235, *F* = 252.57, *p* = 4.11 × 10^−39^) and by a significant interaction “LINE1/LUMA” by “data subset” (*df* = 2,235, *F* = 823.72, *p* = 6.62 × 10^−107^). The differences were statistically significant for all data subsets (Wilcoxon signed rank tests: all *p* value <0.001, Table 2). Additional visual inspection of the scatterplot of the LINE-1 versus the LUMA measurements (Fig. 2) indicated that only data acquired in SHSY5Y cells was scattered around the line of equality while samples acquired in MCF7 cells were located below and those acquired in human blood samples above that line. Finally, a statistically significant correlation of the DNA methylation between the two assays was observed in all data subsets (Table 2), however, only weakly in blood samples.

### Method agreement and bias

Applying the method of 95 % limits of agreement identified significant bias between the results obtained with the two bioassays. This was observed as a deviation from zero of the differences between the DNA methylation measured using LINE-1 and that measured in the same sample using LUMA (Fig. 3). The deviation of that difference from zero was statistically significant in all data subsets (one-sample *t* tests: *p* < 0.001, Table 2). Differences between assay results were normally distributed as indicated by non-significant Kolmogorov-Smirnov tests. A fixed bias between the results obtained with the two assays was observed, that is, the deviation of that difference from zero was consistent across the observed range of DNA methylation, i.e., the slope of a regression line through this difference did not significantly differ from zero (Table 2) indicating that the bias between both measurements was independent form the degree of methylation.

### Pattern of interassay differences

Interassay differences among human blood, MCF7 cells, or SHSY5Y were large enough to render the LINE-1-LUMA difference in DNA methylation as a good predictor of tissue origin. The multimodal distribution of the differences could be described by a mixture model with *M* = 3 Gaussians (Fig. 4). Bayesian decision limits of −6.3 and 12.3 % DNA methylation were observed. Different data subsets were unequally represented among the Gaussians (*χ*^2^ = 299.67, *df* = 8, *p* < 2.2 × 10^−16^). This provided a basis to build a decision-tree algorithm (Fig. 4) that was able to predict from the originating tissues (data subsets) in which Gaussian an interassay difference will be placed at a cross-validated accuracy of 83.6 %.

### Linear regression

Finally, linear regression analysis was used to further characterize deviations of the results obtained from both assays from the line of identity (Fig. 2). The approach employed goodness-of-fit-based statistics to substantiate tissue-dependent deviations of the linear model from a y-intersection of zero and a slope of a value of one (Eq. 3). The goodness of fit was greatly improved when allowing separate parameter values for each data subset (Δ_−2LL_ = −532.877, *p* < 0.0001). However, this was not statistically supported for every data subset, and when allowing certain subsets to share parameter value, the fit was not always worsened.

The final model indicated the following results (Table 3). Firstly, the two in vitro cell lines differed with respect to their y-intercepts, i.e., the y-intercept for the MCF7 cell line was zero while that for the SHSY5Y cell line was located at 9 % and that for the blood cells was located at 71.1 % DNA methylation. This was statistically supported by a non-significant increase in −2LL when the intersection parameter was fixed at a value of 0 for the MFC7 cell line (*θ*_Int,Subset1_; Δ_−2LL_ = +1.178) but a significant increase when the same was done for the SHSY5Y cell line (*θ*_Int,Subset2_; Δ_−2LL_ = +97.882; *p* < 0.0001) or for the blood cells (*θ*_Int,Subset3_; Δ_−2LL_ = + 445.892; *p* < 0.0001). Secondly, the final linear regression model indicated that the two cell lines shared the same slope of 0.8978 as −2LL raised only non-significantly by 0.02 (*p* > 0.05) when setting *θ*_Slope,Subset1_ equal to θ_Slope,Subset2_ while associating the same slope also to the blood cells worsened the fit. Thus, the results of the regression analysis indicated differences among tissue types consisting of (i) the cell lines shared the same slope but had a significantly different y-intercept and (ii) the blood sample differed from the relationship observed in the cell lines with respect to both y-intercept and slope of the linear relationship between LINE-1- and LUMA-derived measurements of global DNA methylation.

**Table 3:** Parameters and estimated values of the final linear model of the data-subset specific relation of LINE-1 and LUMA assay-based measurements of global DNA methylation. The full model was given as Methylation_LINE1_ = (Intersection + *θ*_Int, Subset1.. Subset3_) + (Slope × *θ*_Slope, Subset1.. Subset3_) × Methylation_LUMA_ + *ε*, where Intersection and Slope are structural parameters of the linear model denoted during the fitting as *θ*_1_ and *θ*_1_, respectively, *θ*_Int,Subset1.. Subset3_ and *θ*_Slope, Subset1.. Subset3_ are data subset specific modulators of the structural parameter values, denoted during the fitting as *θ*_3..12_, and *η*_1_ and *ε* accounts for the additive error in the fit of the percent methylation data acquired by means of two different assays. The final model was the result of the model building favoring the best but sparsest model based on goodness-of-fit statistics

Parameter	Value (and % SEE)	95 % bootstrap CI
Y-intersection (% methylation) = *θ*_1_	0 (fixed)	–
*θ*_3_ = *θ*_Int,Subset1_	0 (fixed)	–
*θ*_4_ = *θ*_Int,Subset2_	9.03 (9.5)	(7.3, 19.9)
*θ*_5_ = *θ*_Int,Subset3_	71.1 (8.4)	(58.7, 87)
Slope = *θ*_2_	0.38 (1.1)	(0.32, 0.4)
*θ*_6_ = *θ*_Slope,Subset1_	θ _6_ = θ_7_ = 2.68 (0.7)	(2.2, 2.8)
*θ*_7_ = *θ*_Slope,Subset2_	Slope_Subset1,2_ = 2.68 · 0.38 = 0.9	
*θ*_8_ = *θ*_Slope,Subset3_	0.88 (27.7)	(0.2, 1.3)
	Slope_Subset3_ = 0.88 · 0.38 = 0.29	

*SEE* standard error of parameter estimate, *fixed* the parameter was not estimated but set at the shown value, *CI* 95 % confidence interval of parameter estimate, obtained from 1000 model runs of the final model with bootstrap resampled data

## Discussion

Different approaches applied to the agreement of global DNA methylation measured by LINE-1 and LUMA in three different DNA sample subsets consistently rejected the assumption of complete agreement between the two bioassays (Fig. 1). Moreover, the differences between the two assays were tissue-dependent.

The disagreement of the two assays seems biologically plausible as the two assays pursue different basic approaches not necessarily leading to the same picture of global DNA methylation. LINE-1 and LUMA differ with respect to their CpG recognition sites at the DNA. Specifically, DNA methylation occurs to 70–80 % of cytosines that locate within CpG dinucleotides [71]. This corresponds to 3–5 % of all cytosines of the human genome [72]. CpG dinucleotides are enriched in CpG islands, repetitive sequences, and CpG island shores [73] and in approximately 60 % of all gene promoters [74, 75]. The LINE-1-based assay selectively measures the methylation of CpG islands located within long interspersed nucleotide elements (LINE). These have a length of up to 6 kb and with >500,000 copies account for approximately 17–20 % of the human genome [19–21]. However, LINE-1 is unevenly distributed throughout the genome [76], and in addition, most of them are excluded from genomic regions containing housekeeping genes [77]. LINE-1 is most frequently methylated in somatic tissues, where an estimated one third of DNA methylation occurs in these repetitive sequences [78] and particularly dense in X chromosomes [17, 25]. By including four CpG positions within the LINE-1 sequence, the pyrosequencing assay recognized 2,000,000 CpG sites when 500,000 copy numbers are estimated through the genome, i.e., roughly 7 % of the whole CpG dinucleotide contents of the human genome. However, not all LINE-1 elements are of full length; most of them are truncated and just about 10,000 LINE-1 elements contain a 5′UTR. Therefore, the effective recognized CpG dinucleotide content should be lower than 7 % [79].

In contrast to the LINE-1-based assay, the LUMA method measures the DNA methylation also outside repetitive elements [22]. However, the target sequence CCGG of its restriction enzyme HpaII does also not cover all CpGs. Of the 28,000,000 CpG dinucleotides in the human genome, 4.14 % are within HpaII target sites (CCGG) located in repetitive elements and 3.90 % in unique sequences [25]. HpaII covers 11.7 and 12.9 % of CpGs in promoter and CpG islands, respectively [80]. C+G-rich regions of the genome have been associated with increased gene numbers [81, 82], higher amounts of CpG islands [83], and enhanced transcriptional activity [82]. HpaII target sites are 15-fold enriched in CpG islands so that analysis of HpaII digested sites may over-represent potentially important regulatory sequences [25, 84]. This is a further contrast to LINE-1 sequences, which are enriched in A+T-rich gene regions [85] associated with fewer gene numbers and a lower transcription rate. Furthermore, from their target sequences across the genome, LINE-1 and LUMA may indeed measure different DNA methylation facets of epigenetic regulation of gene expression. This makes the consistently observed disagreement between them as biomarkers of DNA methylation biologically plausible.

The biological differences between LINE-1 and LUMA may add technical differences of the assays. Specifically, the CpG sequence targeted by LINE-1 pyrosequencing in the 5′ region tends to be deleted at unknown frequency. Approximately 2000 of the LINE-1 elements are active [76] that can reintegrate into the human genome results in generation of new LINE-1 sequences. Therefore, the count of the analyzed elements is unstable and may vary among different samples and individuals [17]. Moreover, primers should amplify the region of interest regardless of its methylation status, but in practice, complete independence of the methylation is often not achievable due to a PCR bias favoring amplification of unmethylated templates [86]. This may explain why the methylation of the completely methylated controls was not quantified as 100 % by the LINE-1-based assay (data not shown). This resembles observations with this assay in other laboratories [18]. Although linearity of the calibration curve between non-methylated and completely methylated controls allowed for a valid recalibration of the results, the difference to the LUMA assay that quantified the completely methylated control closer to 100 % (95.7 %) is a factor contributing to the dissimilarity between the methods.

**Fig. 5:**
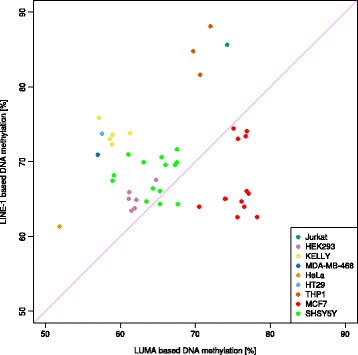
*Scatterplot* of measurements of global DNA methylation. In several different cell lines, the LINE-1 (ordinate)- and the LUMA (abscissa)-based bioassays were applied to quantify global DNA methylation. The *solid magenta line* marks identity

The analyses also pointed at a tissue dependency of the degree of agreement between the LINE-1- and the LUMA-based measurements of DNA methylation. This agrees with independent evidence of tissue-specific differential DNA methylation among 17 human somatic tissues [87]. To further strengthen the observation of a cell population-dependent effect, additional measurements were performed assessing the DNA methylation level in further seven cell lines using the two assays. This additional set of samples comprised a mixture of HEK 293 (human embryonic kidney), KELLY (human neuroblastoma cells), Jurkat (human T lymphocytes), MDA-MB-468 (human mammary gland/breast cancer), HeLa (human cervical cancer), HT29 (human colon carcinoma), and THP1 (human monocytes from acute monocytic leukemia) cells. Following laboratory assays, the DNA methylation data obtained with the LINE-1 were plotted against those obtained with the LUMA method (Fig. 5). This scatterplot showed that the location of most data points was not on or close to the line of equality. Hence, differences between LINE-1- and LUMA-based measurements of global DNA methylation seem to be a consistent observation in various cell or tissue types, not restricted to the tissue types chosen for the present main analyses.

As in the present analysis, human blood was used as a frequent material in clinical epigenetic studies [26–36]; it is important to note that the heterogeneous composition of different blood cells within whole blood samples has an influence on the determined global methylation [37, 88, 89]. Therefore, associations of global methylation patterns with certain health-associated conditions, such as with inflammatory diseases, can be the result of a cell composition effect. To avoid such influences, the use of better-defined cell population should be considered. Moreover, the DNA extraction method can influence the measurement of global DNA methylation. In the present case, however, both assays were run on the same DNA samples, which reduces the probability that the differences between LINE-1- and LUMA-based readouts were caused by cell composition effects within blood samples or by an extraction bias.

**Fig. 6:**
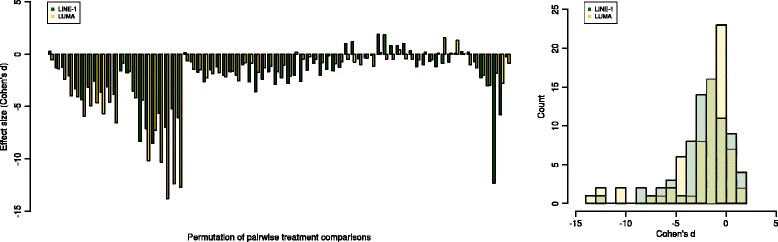
Overview about the effect sizes, calculated as Cohen’s *d*, obtained when using the LINE-1 or the LUMA approach to the quantification of global DNA methylation. A total of *n* = 72 effect sizes was calculated from a comparison of every treatment with every other treatment to which the cell lines had been exposed (Table 2; DMSO and untreated conditions combined). The *bar plot* (*left*) shows the effect sizes that would be obtained when using either the Line-1 (*green*) or the LUMA (*gold*) method in pairs for each paired comparison between two treatments. The *histogram* (*right*) shows the high degree of superposition of the effect sizes that would be obtained when applying the two assays

Finally, while the statistical analyses and biological reasoning provided support for rejecting an agreement between the two common biomarkers of global DNA methylation, the present method comparison cannot provide a choice of the better method as a test against the gold standard as provided by an HPLC analysis. Since the gold standard HPLC specifies the DNA methylation level as the percentage of 5-^m^C relative to the whole cytosine amount of genomic DNA, the surrogate markers LINE-1 and LUMA specify the DNA methylation as the percentage of 5-^m^C in CpG dinucleotides within the recognition side. Therefore, a comparison of absolute values with the true value from HPLC analysis is not possible. Moreover, the statistically significant correlation between the results of the two assays in all data subsets (Table 2) supported the utility of both methods as global methylation markers. Indeed, an exploration of the effects sizes, estimated as Cohen’s *d* [90], produced by the various treatments to which the two cell lines were exposed supported the suitability of both methods to assess changes in DNA methylation. Specifically, a permutation approach provided a total of 72 paired comparisons between all different treatments (66 in MCF7 cells and six in SHSY5Y cells). The values of Cohen’s *d* [90] calculated for the effects resulting when the LINE-1 based assay was used, and again, when the LUMA method was used, it indicated comparable effect sizes (Fig. 6) and were significantly correlated (Spearman’s *ρ*: 0.79, *p* < 0.0001). As the sample sizes were often very small, the numerical results of this accessory analysis have, however, to be interpreted with caution.

## Conclusions

Different approaches to the agreement of LINE-1- and LUMA-based measurements of global DNA methylation were applied to three human-derived cell types, and the assumption of complete agreement between the two bioassays were consistently rejected. Although providing partly correlated measurements of DNA methylation, interchangeability of the quantitative results obtained from the two methods was jeopardized by a consistent bias between the results. Moreover, present analyses strongly indicate a tissue specificity of the differences between the two methods.
